# Predicting the establishment success of introduced target species in grassland restoration by functional traits

**DOI:** 10.1002/ece3.3268

**Published:** 2017-08-11

**Authors:** Karina Engst, Annett Baasch, Helge Bruelheide

**Affiliations:** ^1^ Institute of Biology/Geobotany and Botanical Garden Martin Luther University Halle‐Wittenberg Halle Germany; ^2^ Department for Nature Conservation and Landscape Planning Anhalt University of Applied Sciences Bernburg Germany; ^3^ German Centre for Integrative Biodiversity Research (iDiv) Halle‐Jena‐Leipzig Leipzig Germany

**Keywords:** functional traits, Germany, grassland restoration, hay transfer, on‐site threshing, seeding, seedling establishment, species introduction

## Abstract

Species‐rich semi‐natural grasslands are highly endangered habitats in Central Europe and numerous restoration efforts have been made to compensate for the losses in the last decades. However, some plant species could become more easily established than others. The establishment success of 37 species was analyzed over 6 years at two study sites of a restoration project in Germany where hay transfer and sowing of threshing material in combination with additional sowing were applied. The effects of the restoration method applied, time since the restoration took place, traits related to germination, dispersal, and reproduction, and combinations of these traits on the establishment were analyzed. While the specific restoration method of how seeds were transferred played a subordinate role, the establishment success depended in particular on traits such as flower season or the lifeform. Species flowering in autumn, such as *Pastinaca sativa* and *Serratula tinctoria*, became established better than species flowering in other seasons, probably because they could complete their life cycle, resulting in increasingly stronger seed pressure with time. Geophytes, like *Allium angulosum* and *Galium boreale*, became established very poorly, but showed an increase with study duration. For various traits, we found significant trait by method and trait by year interactions, indicating that different traits promoted establishment under different conditions. Using a multi‐model approach, we tested whether traits acted in combination. For the first years and the last year, we found that models with three traits explained establishment success better than models with a single trait or two traits. While traits had only an additive effect on the establishment success in the first years, trait interactions became important thereafter. The most important trait was the season of flowering, which occurred in all best models from the third year onwards. Overall, our approach revealed the potential of functional trait analysis to predict success in restoration projects.

## 1 INTRODUCTION

Restoration projects that aim at restoring unique species compositions, biodiversity, and functionality (Kiehl, Kirmer, Donath, Rasran, & Hölzel, 2010; Laughlin, 2014; Török, Vida, Deák, Lengyel, & Tóthmérész, 2011) are applied across numerous terrestrial habitats, especially in semi‐natural grasslands. To understand the ecological mechanism of restoration success, it is of great interest to know which species can become established while others cannot and which are the main drivers for successful species‐specific establishment. It is well known that some species are more difficult to become established than others (e.g., Bischoff, 2002), indicating that successful establishment depends on characteristics of the target species. It is clear that not all species can become established as the species’ niches have to overlap with the site conditions at a particular location (“*environmental filtering*”, Kraft et al., 2015). Successful colonizers also have to avoid competitive exclusion by co‐colonizers that were able to establish themselves simultaneously (“*biotic filtering*”, Belyea & Lancaster, 1999; Mayfield & Levine, 2010; Kraft et al., 2015). From the species perspective, certain conditions for successful germination and establishment have to be fulfilled (“*regeneration niche*”, Grubb, 1977; “*safe sites*”, Harper, Williams, & Sagar, 1965; Harper, 1977). Furthermore, successful establishment might require different functional traits defined as a species’ morphological, biochemical, physiological, structural, phenological, or behavioral attributes relevant to the species’ response to the environment (Violle et al., 2007; Díaz et al., 2013). In the context of restoration, functional traits affect different stages of a species’ life cycle such as germination, dispersal, and persistence. For instance, a low seed mass or a high seed number might increase the probability to reach a safe site. A high seed mass has been identified as being pivotal for establishment success (Fischer, Von der Lippe, Rillig, & Kowarik, 2013; Leishman, Wright, Moles, & Westoby, 2000; Westoby, Leishman, & Lord, 1996), which provides the resources in the germination phase (Cornelissen et al., 2003; Westoby et al., 1996), but which trades off with seed number (see Leishman, 2001). Traits conferring competitive ability such as a high SLA or tall stature prevent competitive exclusion and are linked to biotic filtering (Mayfield & Levine, 2010), which might be important for the species persistence. For these reasons, it is unlikely that the establishment success of a species in a restoration project depends on a single trait only. Although it has been demonstrated that different traits are highly coordinated (Westoby & Wright, 2006), as exemplified by the leaf economic spectrum (Wright et al., 2004), many different trait dimensions are required to explain a species’ regeneration niche (Grubb, 1977). Despite the importance of different trait dimensions, approaches that detect the effect of trait combinations are rare (e.g., Küster, Kühn, Bruelheide, & Klotz, 2008). Nevertheless, it can be expected that a certain trait state is only of advantage for a plant if a certain state of another trait is also given, thus forming an ecological strategy that enable the species to survive, reproduce, and become established (Donovan, Maherali, Caruso, Huber, & De Kroon, 2011). For example, in a restoration project the trait of a high seed production may only be advantageous if the generative stage is reached quickly after germination. Mechanistically, trait interactions should matter as successful establishment is the outcome of several transition probabilities in a species’ life cycle. The overall outcome of establishment is multiplicative. Thus, two or more low transition probabilities can co‐limit the successful establishment of a species. In consequence, trait states should increase establishment only if acting in concert rather than as soloists.

In restoration projects, species establishment does not only depend on environmental conditions and species traits but also on the restoration methods applied. Apart from spontaneous colonization, one aspect is that species are often deliberately introduced to restore species‐poor grasslands (see Kiehl et al., 2010; Török et al., 2011). The techniques of species introduction comprise, among others, transfer of freshly cut seed‐rich hay (e.g., Baasch, Kirmer, & Tischew, 2012; Edwards et al., 2007; Klimkowska, Van Diggelen, Bakker, & Grootjans, 2007; Schmiede, Otte, & Donath, 2012), transfer of diaspores extracted from fresh hay by on‐site threshing (Scotton, 2016; Scotton, Kirmer, & Krautzer, 2012), sowing of regional seed mixtures (Kirmer, Baasch & Tischew 2012; Lepš et al., 2007; Prach, Jongepierová, & Řehounková, 2013; Warren, Christal, & Wilson, 2002). or a combination of several of these measures (Baasch, Engst, Schmiede, May, & Tischew, 2016; Engst et al., 2016; Török et al., 2012). In general, different types of restoration techniques may differ in overall establishment success of introduced species as Scotton et al. (2012) reported a seed yield for freshly cut hay of nearly 100%, while seeds from threshing yielded only 50%–80%.

Finally, the importance of all factors mentioned so far vary with time. In general, many restoration projects report increasing establishment success with time (Pywell et al., 2007; Scotton, 2016). In the first years, species have to quickly germinate to leave the seedling stage, which probably is facilitated by a large diaspore size (Leishman et al., 2000; Westoby et al., 1996). As more and more individuals can establish themselves, competition intensity increases and traits conferring competitive strength, such as high SLA and tall stature can be expected to become more important with time.

We analyzed these aspects in a grassland restoration project at two species‐poor meadows where the establishment success of 37 species was monitored for 6 years. We hypothesized (1) that across both restoration methods and time steps the successful establishment of species depends on traits that are related to seed dispersal and germination. In contrast, traits related to persistence (such as clonal spread) and competitive ability are likely of minor importance, particular in the early stages of the restoration. In addition, we tested for the effect of design‐specific variables that resulted from our experimental set‐up. We expected that the information whether a species was present at the donor site or in the seed mixture increases the predicted establishment success. Furthermore, we assessed the context‐dependency of traits for (2) different restoration methods and (3) points in time, by testing for interactions of traits with these factors. Finally, we hypothesized that (4) interactions between different traits explain establishment success better than single traits alone or additive effects of several traits. In particular, we expected that traits related to dispersal and germination disproportionally contribute to establishment success if a species combines favorable states in these traits simultaneously.

## 2 MATERIALS AND METHODS

### 2.1 Study area and species selection

We employed species data of a grassland restoration project at two study sites, “Küchenholzgraben” and “Untere Schwarze Elster,” that was conducted in Saxony‐Anhalt, Germany to introduce target species by different methods in species‐poor grassland. At both sites, the implementation of restoration measures took place in 2009 and the same strip‐design was applied (see Baasch et al., 2016; Engst et al., 2016). Six randomized blocks were established, each measuring 6 m × 120 m and subjected to two crossed treatments, transfer of seed‐containing plant material and sowing of regional seed mixtures (four variants). Transfer of seed‐containing plant material was applied by (1) freshly cut hay (H) and (2) seeds extracted from fresh hay by on‐site threshing (T). These two types of seed addition methods were combined either with or without additional sowing of regional seed mixtures. In total, at each site we had 24 plots (2 × 2 treatments × 6 blocks). Before the seed‐rich plant material was applied, the soil at the establishment strips was tilled and rolled. The donor sites for hay and threshing material were different in the two study sites, and thus, differed in species that could be transferred, but in both cases, they were located near to the respective receptor sites. Similarly, seeds in the additional seed mixtures differed between the two sites (Table S1). The seeds for addition of seed mixtures were provided by a wildflower seed producer, grown from regional provenances (Table S1). We monitored the vegetation annually from 2010 to 2015 on 4 m × 4 m plots and recorded percentage cover for every species. In addition, the receptor and donor sites were also surveyed in 2009 before the restoration started. The detailed results of the restoration monitoring at both study sites have already been reported (see Baasch et al., 2016 and Engst et al., 2016). For this paper, we addressed a more specific issue, thus using only a part of the entire data set. We included only those plots that were treated with an additional sowing (*n* = 12 per site) to make sure that all analyzed plots within one site received all the same species. Furthermore, for both sites only target species were selected that could have been potentially introduced by the applied restoration methods and that were not present at the receptor sites in 2009. Therefore, we considered only those species that were either present at the donor site plots in 2009 or were included in the regional seed mixtures, resulting in a total pool of 37 species (see Table S1).

### 2.2 Plant traits

We selected traits associated with the response to species establishment based on germination, dispersal, and persistence (Tables S2 and S3). Dispersal traits were fruit type, diaspore type, heterodiaspory, exposure of diaspores, diaspore morphology, diaspore form, seed shape index (length:width ratio) as well as season of flowering, and seed shedding. Traits linked to germination were seed mass, seed number, season of germination, and dormancy. For comparison with these traits related to establishment, we included traits describing the competitive ability such as height and growth rates such as SLA and LDMC. Furthermore, we included persistence traits such as lifeform, Grime's (1979) competitor–stress‐tolerator–ruderal (CSR) scheme, rosette type, age of first flowering, and flower duration. Finally, we used the type of clonal growth organs (CGO), as a long‐term restoration success also relies on keeping the once germinated plants in the long run. We used all CGO's listed as obligate in the CLO‐PLA database. In case of conflicting information on CGO's, we followed the data of Schubert (Schubert in Klimešová & De Bello, 2009). All categorical traits were 0/1 coded. As far as possible, we tried to achieve a balance between the different categories within one trait by fusing highly under‐represented categories into meaningful groups that comprised at least three species, except for lifeform (see Table S2 for further explanation of traits included and groups formed). For *Poa pratensis*,* Poa trivialis,* and *Lolium perenne* we only used diaspore type fruit. Flower season in summer and CGO bulb were excluded from the analyses, as these traits were found in just one species each. The numeric traits SLA, LDMC, height, and seed weight were obtained from different data sources (Table S2). To account for systematic measurement biases between these different data sources, we performed linear regressions based on a large grassland‐species data set on numeric trait values of species common to two different data sources and then applied these regressions to species only present in one of the databases when combining trait values from both sources.

### 2.3 Statistical analysis

For each site, we calculated the relative introduction rate of target species as the number of species found during the whole observation period divided by the total number of introduced species in 2009 to show the overall potential of the applied restoration methods. Spearman‐correlation coefficients were used to test for intercorrelations among plant traits and for highly correlated traits such as plant height and releasing height just one was used. To test the hypothesis that plant species traits affect the establishment success of the species, we performed a two‐step approach. In the first step, we modeled the occurrence of the species as a proxy for their establishment success with linear mixed effect models across all years at the plot level, using the presence of each species in each of the 12 plots per site as response variable and a logit link function with binomial error distribution. We used the GLIMMIX procedure of SAS (SAS Institute Inc., Cary, NC, USA), approximating maximum likelihood with Laplace's method and deriving the degrees of freedom of the error terms with the containment method. Fixed factors were (1) restoration method and year, as well as their interaction, while the species, block, plot, and site were included as random effects (no‐trait model). Year entered the model as continuous variable. Afterward, to omit noninformative traits, we included trait by trait (2) species traits as further fixed factor, keeping all other components of the preceding model (1) (single‐trait models), except replacing species as random factor with species × trait interaction, which has identical levels as species because traits did not vary within species. However, we used the species × trait interaction instead of species only to inform SAS that traits have to use species as error term, thus making sure that the correct degrees of freedom were obtained in the subsequent ANOVA tables. However, there was no difference in the amount of variance explained by species × trait interaction and species. We also calculated models with design‐specific variables that described (3) the presence of a species at the donor site, or (4) the presence in the seed mixture and treated this variable like a trait, also keeping all other components of the preceding model (2). The significance of the predictors was tested with type III ANOVA. All models were ranked by Akaike's information criterion (AIC) and all models of (2)–(4) performing better than that of (1) (the no‐trait model) were subsequently examined for effects.

In a second step, we analyzed the combined effect of traits on establishment at the species level. Principal component analysis (PCA) was employed to identify the main gradients of trait states and trait values in all species. Post hoc correlations were used to relate PCA axis scores to the establishment rates of every species averaged across restoration methods for each of the observation years. The analyses were performed using the vegan package (Oksanen et al., 2015) in R. We also used the averaged establishment rates to analyze the combined effect of several species traits. Across all treatments and blocks (i.e., all 12 plots per site), we counted the number of plots in which each species occurred and the number of plots in which they were not present, separately for all years and applied generalized linear models with logit link function to relate establishment to the traits. For those species that occurred only at the donor site or in the seed mixture of one of the two study sites, the number of plots in which a species could be potentially present was 12. For those species that occurred at the donor sites or in the seed mixtures of both study sites, the number of potentially colonizable plots was 24. We employed a two‐step model selection procedure, separately for all years. In a first selection step, we disregarded variable interactions and included all two‐variable combinations for those variables that showed a smaller AIC in step 1 than the no‐trait model and ranked all models by AICc (Akaike's Information Criterion corrected for small sample sizes). We then identified the seven variables that were included in one of the top models, starting with the best model and stopping when the number of seven variables was reached. In a second step, we additionally included variable interactions of all these seven variables and then identified the best three‐predictors model (multitrait model) according to AICc, with either three different variables or two variables and their interaction. The coefficient of determination as a measure of model fit was calculated as one minus residual deviance divided by null deviance (1‐residual deviance/null deviance). This procedure was conducted separately for all years and applied to models containing traits. The analyses were performed using the glm procedure in the stats package (R Core Team 2015) and the dredge procedure in the MuMIn package (Bartoń, 2016).

## 3 RESULTS

### 3.1 Success of introduced target species at the site level

There were large differences in the degree of occurrence among species (see Table S1). On the one hand, some species had a high frequency of occupied plots (e.g., *Achillea ptarmica* or *Knautia arvensis*). On the other hand, there were also species that could not establish themselves at all or only in single plots (e.g., *Pimpinella major, Ranunculus auricomus* agg. or *Symphytum officinale*). During the whole study period, 17 of 19 and 18 of 31 introduced target species were recorded in the two study sites “Küchenholzgraben” and “Untere Schwarze Elster,” respectively, resulting in relative introduction rates of 90% and 60%, respectively.

### 3.2 Establishment success related to species traits

**Figure 1:**
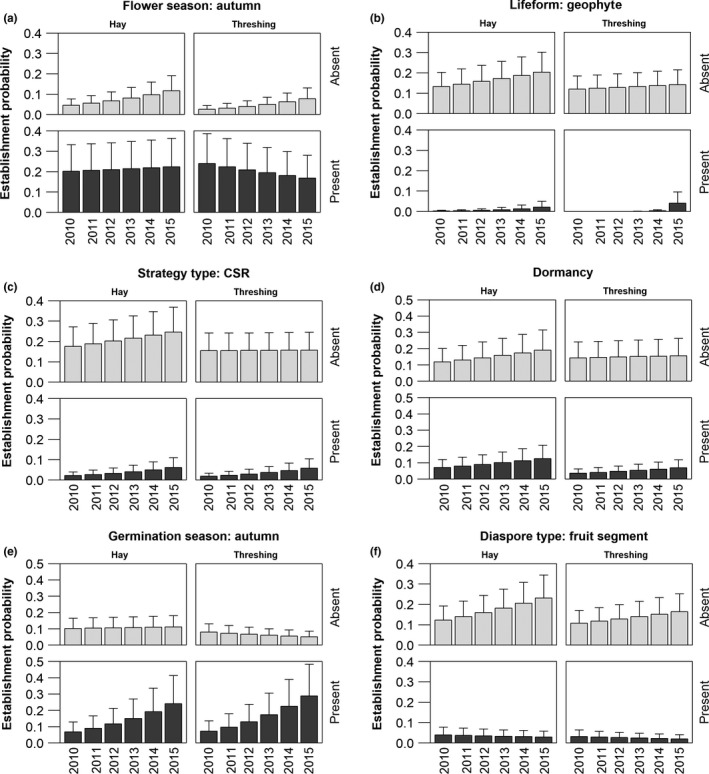
Establishment probability of species depending on species traits and the method applied (hay: hay transfer, threshing: transfer of threshing material). The graphs show six models of key traits from Table 1. (a) flower season, (b) lifeform, (c) strategy type, (d) dormancy, (e) germination season, (f) diaspore type. Present and absent = the trait state was present or absent in the target species, respectively

Testing the establishment success in single‐trait models at the plot level, 24 models of 49 models ranked higher than the no‐trait model (Table 1, for all models see also Table S5). Five traits had a significant main effect on the establishment success irrespective of restoration method and time (life form: geophyte, life form: hemicryptophyte, flower season: autumn, ecological strategy: CSR, diaspore morphology: flat appendages). For instance, species flowering in autumn (e.g., *Achillea ptarmica*,* Pastinaca sativa*, or *Serratula tinctoria*) were able to establish themselves better (Figure 1a) than species flowering in other seasons (e.g., *Ranunculus auricomus* agg. or *Cynosurus cristatus*). Species that were not geophytes became more easily established. Geophytes, such as *Allium angulosum* or *Galium boreale*, showed a very low establishment probability in particular in the first years, which increased with time (Figure 1b). The same pattern was found for the CSR‐strategists. Establishment was higher if the species did not belong to the “CSR‐strategy” type (Figure 1c).

**Table 1:** ANOVA results of linear mixed effect models for the response of successful establishment of species depending on one predictor variable, method, year, and their interactions

Variable	Rank	AIC	Variable	Method	Variable* method	Year	Variable* year	Year* method	Variable* year* method
*Design‐specific variables*									
Presence at donor site	16	2,535.26	0.40	0.05	0.25	11.84***	4.07*	0.83	0.06
Presence in the seed mixture	1	2,481.77	8.21**	0.52	4.39*	5.27*	0.33	0.10	3.33^.^
*Traits*									
Germination season: autumn	2	2,506.75	0.07	0.04	0.63	18.18***	29.03***	0.36	1.47
Life form: geophyte	3	2,519.16	9.81**	3.71^.^	3.69^.^	8.62**	7.17**	3.67^.^	4.15*
Germination season: spring	4	2,520.72	0.04	0.05	0.32	14.37***	19.89***	0.51	1.06
Life form: hemicryptophyte	5	2,524.12	5.46*	3.76^.^	3.74^.^	8.71**	7.26**	3.71^.^	4.19*
Flower season: autumn	6	2,524.48	5.03*	0.12	4.58*	9.18**	16.44***	0.46	1.42
Diaspore morphology: flat appendages	7	2,524.61	4.62*	0.03	5.7*	6.58*	5.77*	0.55	0.06
Flower season: spring	8	2,524.73	2.20	0.35	7.00**	3.78^.^	8.82**	0.26	1.11
Seed shedding season: autumn	9	2,524.90	0.52	0.74	1.80	26.83***	18.81***	1.12	4.03*
Germination season: summer	10	2,525.47	0.09	3.97^.^	10.75**	9.10**	2.71	0.78	2.19
Fruit type: explosive release mechanism	11	2,526.36	2.03	0.22	0.18	27.45***	19.49***	0.07	0.62
Diaspore exposure: enclosed	12	2,528.10	1.03	0.18	0.91	18.73***	19.52***	0.19	0.87
Diaspore type: seed	12	2,528.10	1.03	0.18	0.91	18.73***	19.52***	0.19	0.87
Fruit type: nonfleshy fruit	12	2,528.10	1.03	0.18	0.90	18.74***	19.52***	0.19	0.87
SLA	15	2,528.94	1.92	0.06	0.01	13.64***	19.41***	1.75	1.38
LDMC	17	2,535.45	0.00	0.93	1.04	2.68	6.85**	0.48	0.19
Seed number: <1.000	18	2,535.53	2.93	0.10	4.99*	9.32**	5.01*	0.54	1.32
Ecological strategy: CSR	19	2,536.32	6.13*	0.08	0.00	15.1***	6.82**	0.19	0.53
Dormancy	20	2,536.53	1.20	0.21	5.95*	10.35**	1.14	0.41	0.72
Diaspore exposure: covered partly	21	2,536.54	1.45	0.25	2.18	3.02	6.75**	0.44	0.10
Diaspore exposure: exposed	22	2,538.18	0.02	0.01	5.32*	6.01*	3.56^.^	1.17	1.48
Diaspore type: fruit segment	23	2,539.77	1.49	0.10	0.02	0.32	5.82*	0.27	0.02
Diaspore morphology: nutrient‐rich	24	2,540.69	1.84	0.44	4.61*	11.57***	2.19	0.01	2.23
*No‐trait model*	25	2,540.74	–	0.08	–	9.29**	**–**	0.71	–

The table shows all 24 models that were better than the no‐trait model. For the remaining 24 models see Table S5. “Variable” refers to either a particular trait or on the presence at the donor site or in the seed mixture. The models are ranked by AIC. Significant effects are indicated as *p <* *.05,**p <* *.01,***p <* *.001, ^.^ *p *<* *.08, and shown in bold fonts.

The models that included design‐specific variables instead of traits were also better than the no‐trait model, but only presence in the seed mixture ranked higher than any trait. Presence in the seed mixture had also a significant main effect on the establishment success irrespective of restoration method and time. In contrast, the variable presence at the donor site had rank 16 of the best models.

### 3.3 Establishment success related to restoration method and time

The no‐trait model which included only method and year as predictors revealed initially low but significantly increasing establishment probabilities with time (*F*_1,3538_ = 9.29, *p *=* *.0023) for both the hay transfer and the threshing treatments. This temporal increase of establishment was also typical of most models that contained a trait (Table 1). Although we did not encounter a significant main effect of the applied restoration method (hay transfer or threshing) in any model (Table 1), indicating that no restoration method outperformed the other across all species, we identified eight traits that displayed a significant interaction with the restoration method (e.g., dormancy and flower season, Table 1, Figure 1). For instance, species whose seeds exhibited dormancy (e.g., *Silaum silaus* or *Cnidium dubium*) performed better in the hay‐transfer treatment compared to the threshing treatment, whereas in the threshing‐treatment nondormant species performed better (Figure 1d).

Furthermore, 18 traits displayed a significant trait by year interaction, demonstrating that some trait states were less important in the first years and became more important with time, or vice versa. For instance, species that germinated in autumn, such as *Campanula patula* or *Centaurea jacea*, showed an increasing establishment probability with time, while the establishment probability of species germinating in other seasons (e.g., *Pseudolysimachion longifolium*) decreased with study time (Figure 1e).

### 3.4 Establishment success at species level

**Figure 2:**
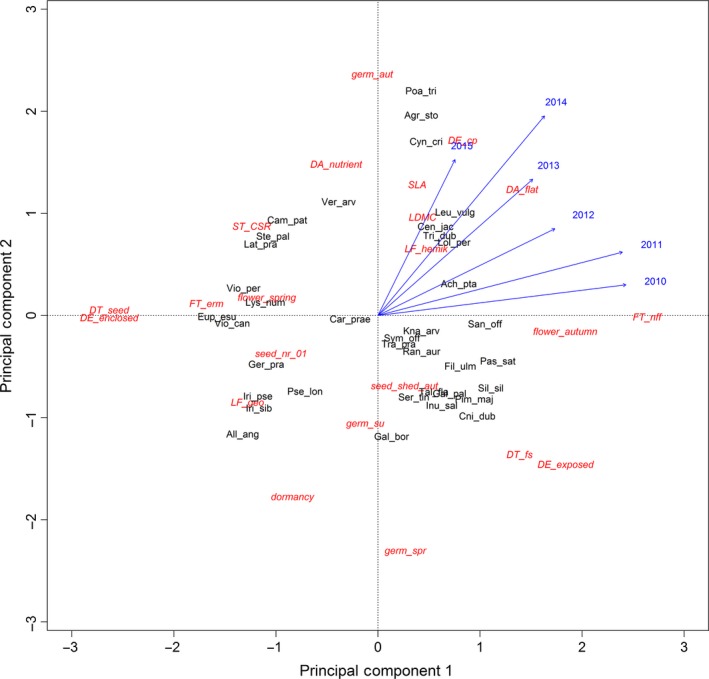
PCA trait‐species biplot representing the trait space of all introduced species (*n* = 37). Trait variances explained by principal components 1 and 2 were 24.2% and 17.5%, respectively. Only traits that ranked higher than the no‐trait model at the plot level (see Table 1) were included (*n* = 22). Trait data were scaled to a mean of 0 and a standard deviation of 1. Blue arrows show post hoc correlations for the establishment rate of each of the species in the respective year. For abbreviation of species and traits see Tables S1 and S2

**Figure 3:**
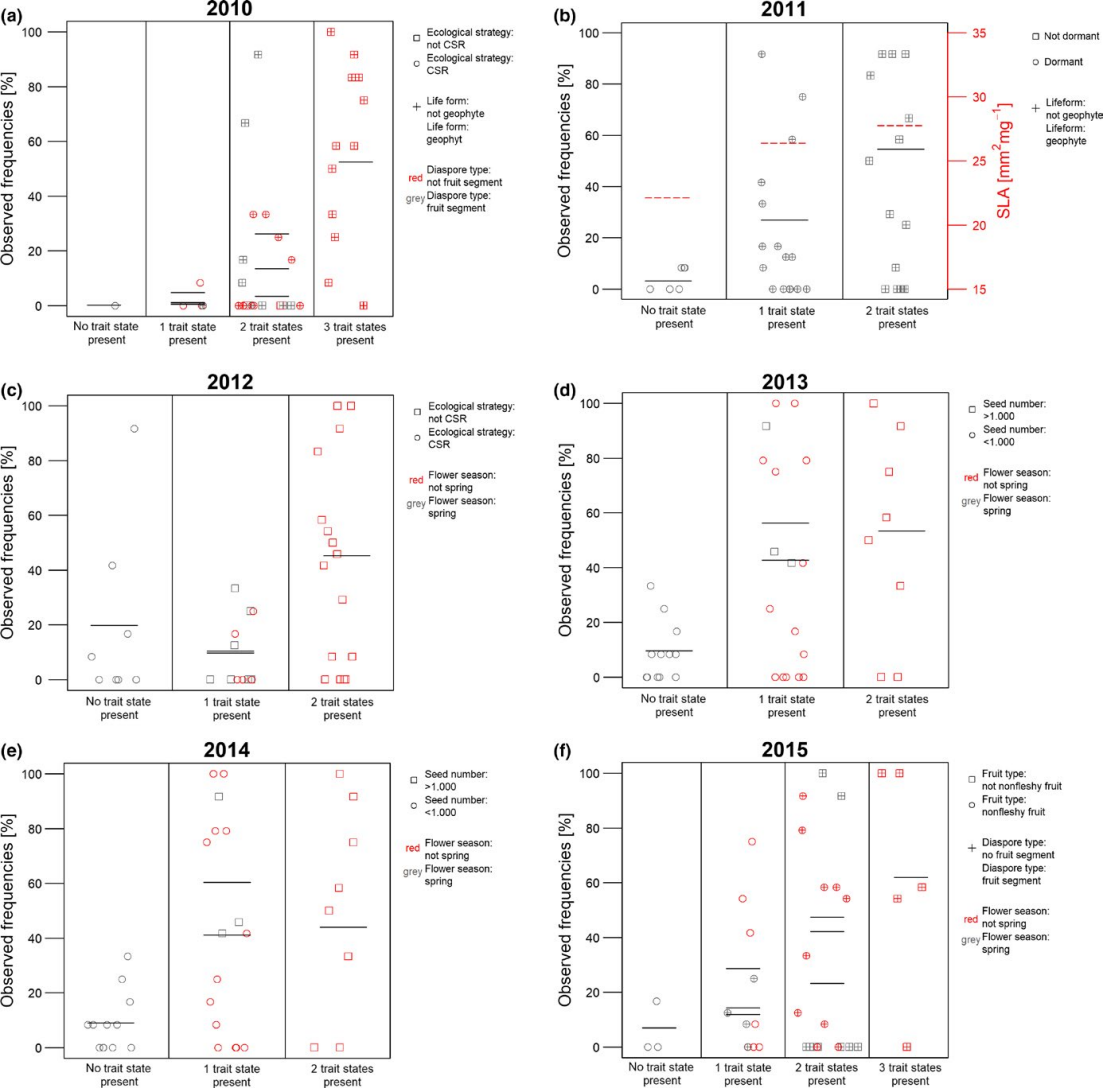
Species × trait plot, representing the best three‐predictors models for every year. Each symbol represents one species (*n* = 37). Horizontal red dashed lines in (b) show the mean values of SLA of species in this group. Horizontal black lines show the establishment probabilities as predicted from the models. For (a) and (f) the three lines in the one‐trait and two‐trait combination columns show all three single traits and all three two‐trait combinations, respectively. For (b)–(e) the two lines in the one‐trait column show all two single traits and the line in the two‐trait combination columns shows the combined effect including their interaction term

At the species level, we analyzed the combined effect of traits on establishment success. The PCA of species traits revealed clear differences between species (Figure 2). We found the establishment success to be strongly correlated with the first PCA axis in the first years and with the second PCA axis in the last years. Traits with high loadings on these axes were mostly already identified as important predictors at the plot level (e.g., germination in autumn, flowering in autumn). The best three‐predictor models are shown in Table 2. For the years 2010, 2011, and 2015, all best models contained combinations of three different traits and outperformed any model that included two traits and the interaction between these traits. In the first year after restoration implementation, the highest establishment success was predicted for those species with a diaspore type different from “fruit segment”, that were no “geophytes” and did not belong to the “CSR‐strategy” type (Figure 3a). Establishment probabilities increased with increasing number of these trait states (Figure 3a), indicating the additive effect of these three traits. As a single trait, a strategy different than CSR had not much effects on establishment success as there were also species with a “CSR‐strategy” that became established well (e.g., *Campanula patula*). However, in combination with the other two traits, not having a CSR‐strategy increased the establishment success considerably, resulting in predicted establishment probability of species characterized by all three favorable trait states to 52% (e.g., *Achillea ptarmica, Geranium pratense,* and *Knautia arvensis*). In contrast, the species that were characterized by none of these three trait states (*Galium boreale*) could establish itself only very poorly. In the second year, being both dormant and a geophyte (e.g., *Allium angulosum* or *Galium boreale*) affected establishment very badly (Figure 3b). From the third year onwards, flowering time in spring turned out to be the most important trait state with negative effects on establishment rates, as this trait occurred in all best models (Table 2). For the years 2012–2014, the best models contained combinations of two different traits including the interaction term of these two traits (Table 2). In 2012, the trait states flowering in spring and CSR‐strategy affected the establishment probability negatively. To increase the establishment probability considerably, both favorable trait states had to be present (Figure 3c). The results for the years 2013 and 2014 showed a slightly different pattern with flower season spring and seed number with <1.000 seeds and their interaction as best model (Table 2, Figure 3d,e). The interaction effect was negative, as one favorable trait state already increased the establishment probability considerably, but two favorable trait states did not result in any further increase. For the establishment in 2015, the best model contained the traits diaspore type, flower season, and fruit type. Establishment success increased for species that exhibited a diaspore type other than fruit segments, a fruit type other than nonfleshy fruits, and that flowered not in spring (Figure 3f). As at the first year, species diaspores consisting of fruit segments, such as *Cnidium dubium* or *Ranunculus auricomus* agg., were less able to establish themselves. The predicted establishment probabilities of the best trait combinations just slightly increased with time and were more or less at 50%. Similarly, the establishment probabilities of the worst trait combinations were never higher than about 10%. Finally, for all years the best models contained combinations of three‐predictor variables and outperformed any model that included only one or two traits.

**Table 2:** The best model predicting establishment success for each of the study years

Year	Best model	AIC	Coefficient of determination
2010	Establishment 2010 ~ Lifeform “geophyte” + Strategy type “CSR” + Diaspore type “fruit segment”	265.76	0.35
2011	Establishment 2011 ~ Dormancy + SLA + Lifeform “geophyte”	284.98	0.27
2012	Establishment 2012 ~ Flower season “spring” + Strategy type “CSR” + Flower season “spring”: Strategy type “CSR”	293.05	0.23
2013	Establishment 2013 ~ Flower season “spring” + Seed number “<1.000” + Flower season “spring”: Seed number “<1.000”	316.18	0.25
2014	Establishment 2014 ~ Flower season “spring” + Seed number “<1.000” + Flower season “spring”: Seed number “<1.000”	299.35	0.25
2015	Establishment 2015 ~ Flower season “spring” + Diaspore type “fruit segment” + Fruit type “non‐fleshy fruit”	338.87	0.19

For the direction of effects see Figure 3.

## 4 DISCUSSION

### 4.1 Species establishment

The relative introduction rates of 60% and 90% observed in our two study sites over the whole observation period are in the mid‐ and upper‐range of success rates reported from other European grassland restoration studies, using hay transfer (21%–80%, Kiehl et al., 2010), sowing of threshing material (41%–77%, Kirmer et al., 2012), or sowing of a regional seed mixture (32%–100%, Kiehl et al., 2010). However, establishment rates varied among species. We demonstrated that differences in establishment success depended on species‐specific traits and on the time elapsed as the restoration measures had been carried out.

### 4.2 Establishment success depending on traits

Across all methods and time steps, the establishment success strongly depended on plant functional traits as 22 models each containing a single trait outperformed the model without traits, thus confirming our general approach of using traits to predict establishment success. Using a combination of three traits in a multitrait model, we were able to explain 35% of the establishment success of species in 2010. Interestingly, the most predictive traits were those related to flowering and not to seed characteristics, thus not supporting our first hypothesis that the most important traits were related to dispersal and germination. The positive impact of flower time in autumn, such as of *Sanguisorba officinalis*, might be explained by allowing a species to complete its life cycle in a meadow that is mown in summer. In contrast, species flowering in spring, such as *Ranunculus auricomus* agg., were at a disadvantage over the whole study period. However, the timing of flowering and seed production has also direct effects on the transfer rates of hay and threshing material. As the restoration measures at both sites had been initiated in autumn, this trait might have resulted in a higher representation of species with that favorable trait state in the transferred seeds. In contrast, seeds of species flowering already in spring or summer might already have been shed at the time of mowing.

While some traits showed significant main effects, promoting establishment under both methods of seed transfer and in all time steps, there was no significant main effect of the applied method. This shows that both transfer methods have been equally successful in terms of the species establishment success. This result is in line with Engst et al. (2016) and Baasch et al. (2016), who encountered only slight differences between both methods applying a trait‐based and species‐based analysis concept, respectively. However, as stated in our second hypothesis we encountered significant trait by method interactions for eight traits. For example, dormant species performed better in the hay transfer than in the threshing treatment. This might be a result of a hay layer brought about by the hay‐transfer treatment in the first year, which may have given seeds more time to become incorporated into the upper soil layers and thus, be subjected to conditions where dormancy was favorable. Our finding of an increasing establishment success with time underlines the statement that complete restoration of grassland communities requires long time scales (Matthews & Spyreas, 2010; Woodcock, McDonald, & Pywell, 2011). The importance of time was also evident in the multitude of trait by year interactions, confirming our third hypothesis. In most of the traits, a certain trait state was less important in the first years but then became more important with time. The overall outcome is the observed increasing number of individuals of introduced target species that were able to establish themselves with increasing study duration. This time‐dependency of traits points to mechanisms in the newly assembled communities, such as the importance of producing numerous seeds or having a high SLA. These traits either increase the reproductive output or confer higher competitive ability, both trait syndromes that become increasingly important in a closed sward (Poorter & De Jong, 1999; Pywell et al., 2003).

Not surprisingly, establishment success also depended on the species’ presence at the donor site or in the seed mixture. It is remarkable that the model with presence at the donor site was outperformed by models based on traits. This may either imply that, irrespective of the local context of a grassland restoration project, species‐specific traits might overrule local constraints, or points to the possibility that the information whether a species was present at the donor site covaried with other, not measured characteristics. We have to consider that this information was purely based on presence/absence qualitative and not on the species’ frequencies at the donor site. We expect that including such quantitative information would have improved our establishment predictions considerably, as a lower abundance at the donor site implies a lower probability to be transferred. In contrast, the presence of seeds in the seed mixture was adjusted and more balanced, which made this information the most important one among all models and highlights the outstanding importance of additionally sowing in restoration projects.

We only found little support for our fourth hypothesis that the establishment success is better explained by interactions between different traits than by single traits alone or by additive effects of several traits. We demonstrated for all years that a three‐predictors model explained establishment success better than models with a single trait or two traits. A study analysing the species’ invasion success in the German flora detected that trait combinations and interactions between traits greatly improved the explanatory power of trait‐based models (Küster et al., 2008). One explanation for not finding interaction effects might be that our set only comprised 37 species compared to 388 species in Küster et al. (2008). With such a low number of species, there is a high probability that in a pair of traits not all combinations of trait states are sufficiently represented, thus precluding any significant interactions. When interactions were included in the best models, they involved traits that were not only related to dispersal and germination but showed an interaction of such traits with flower phenology.

We demonstrated that the explanatory power for establishment success increased with increasing number of favorable trait states. We have to consider that using increasingly more than three states as used by us will finally result in a type of “fingerprinting” of individual species, and at some stage, might make the functional approach obsolete. In our case, each trait combination was still represented by about three species. With fewer or even only one species per trait combination, the functional approach would become a purely taxonomic one, as each species‐specific idiosyncrasy would be also reflected in trait space.

Finally, our results point at the importance of flower phenology rather than of traits related to dispersal, germination, and growth. Across all study years, species flowering in spring and geophytes became established particularly poorly, which leads to two important conclusions. Firstly, both hay making at donor sites and management of the target site have to be more closely adapted to the flower phenology of key target species. At the donor site, such species may be more easily transferred with more than one time of hay making. At the target site, two different mowing regimes alternating in space may increase the establishment success of species that are allowed to finish the life cycle only in one of these two mowing regimes. Secondly, including species with particular phenology, such as those flowering in spring, in additional seed mixtures significantly increases the chance of establishing these species. There are species with unfavorable traits states that could not become established by any of our applied methods (e.g., *Iris sibirica, Pimpinella major*, or *Viola persicifolia*). For such species, practitioners may take into consideration planting them directly into the restoration sites, which has been shown to work amazingly well in managed grasslands (Breitschwerdt, Jandt, & Bruelheide, 2015). Overall, we can conclude that plant species’ traits contribute to improve our understanding of the ecological mechanisms that affect grassland restoration processes. However, a large part of establishment success remained unexplained (at least 65%), which calls for further research. Establishment success may depend on further important traits not included in our study, for example those on germination temperature requirements or germination speed. We also have encountered a strong influence from soft traits, such as strategy type, on establishment, which asks for the underlying hard traits. Finally, establishment success may depend on traits responsible for biotic interactions with other trophic levels. For example, establishment success of such seeds may be decreased that are preferably predated by rodents. Nevertheless, the traits we had included in our study already demonstrated the usefulness of a traits‐based approach in restoration ecology.

## CONFLICT OF INTEREST

None declared.

## AUTHORS’ CONTRIBUTIONS

All authors contributed critically to the drafts and gave final approval for publication. All persons listed as authors meet the criteria for authorship with substantial contributions to: AB: Establishment of the experiment; KE, AB: Data collection; AB, KE, HB: Conception/Research questions of the study; HB, KE: Conception and design of the methodology of the study; KE, HB: Data Analyses and interpretation of data; KE, HB, AB: Writing of the manuscript and revising.

## DATA ACCESSIBILITY

Species trait data have been uploaded as online supporting information Table S3 **.**
