# Supplementary material for: Predicting the establishment success of introduced target species in grassland restoration by functional traits
Source: Ecol Evol. 2017 Aug 11;7(18):7442–53. doi: 10.1002/ece3.3268 (PMC5606862; doi:10.1002/ece3.3268)
Supplement: Supplementary file 1 [file ECE3-7-7442-s001.docx]

Engst, K., Baasch, A. & Bruelheide, H.
Predicting the establishment success of introduced target species in grassland restoration by functional traits

**Supporting Information**

**Table S1.** Information on species presence at the donor site, in the seed mixture and at the receptor site for the years 2010 and 2015 for the study sites ‘Küchenholzgraben’ and ‘Untere Schwarze Elster’. Degree of presence (frequency) is given in classes, showing the percent proportion of plots in which a species occurred: I: <=20%, II: <=40%, III: <=60%, IV: <=80%, V: >80%. Frequencies of species presence at the receptor site are only given for the plots treated with an additional sowing (HS: hay + sowing, TS: threshing + sowing). For detailed information on species presence in all years see Baasch *et al.* (2016) and Engst *et al.* (2016)

**Table S2.** Functional traits used for the analyses related to dispersal, germination and persistence. Categorical traits were coded as binary. To achieve a more balanced distribution of trait states, some categories were fused

| **Trait** | **Description** | **Abbreviation** | **Function** | **Scale**  **(Distribution: 0/1)** | **Main Sources *** |
| --- | --- | --- | --- | --- | --- |
| Fruit type | Characteristics of the fruit: non-fleshy indehiscent fruit; dehiscent fruit (dehiscent fruit with lateral aperture; dehiscent fruit with upright aperture); indehiscent or dehiscent fruits with an explosive release mechanism | FT_nff FT_deh  FT_erm | *dispersal* | binary 13/24 29/8 32/5 | Hintze *et al.* 2013 |
| Diaspore type | Morphological structure acting as diaspore:  seed;  fruit segment;  fruit | DT_s DT_fs DT_f | *dispersal* | binary 24/13 27/10 21/16 | Hintze *et al.* 2013 |
| Heterodiaspory | Species with more than one diaspore types | Dia_heter | *dispersal* | binary 30/7 | Hintze *et al.* 2013 |
| Exposure of diaspores | Accessibility of dispersal vectors to the generative diaspores within the infructescence:  exposed;  covered partly;  enclosed | DE_exp DE_cp DE_encl | *dispersal* | binary 22/15 28/9 24/13 | Hintze *et al.* 2013 |
| Diaspore morphology | Morphology, appendages and structure of diaspores:  nutrient-rich;  aerenchym & mucilaginous surface;  flat appendages;  appendages with hooks & elongated appendages;  no | DA_nutrient DA_ballo_muci DA_flat DA_hook_elon DA_no | *dispersal* | binary 26/11 29/8 26/11 24/13 29/8 | Hintze *et al.* 2013 |
| Diaspore form | Form of diaspore:  spherical | DF_spher | *dispersal* | binary 11/26 | Hintze *et al.* 2013 |
| Seed shape index | Ratio of seed length and seed width | Dia_shape | *dispersal* | numerical | Hintze *et al.* 2013  Otto 2002 |
| Seed shedding season | Season in which seeds fall out:  spring;  summer;  autumn;  winter | seed_shed_spr seed_shed_su seed_shed_aut seed_shed_wi | *dispersal* | binary 34/3 7/30 5/32 31/6 | Kleyer *et al.* 2008  Poschlod *et al.* 2003  Fitter & Peat 1994 |
| Flower season | Season in which plant species flowers:  spring;  autumn | flower_spring flower_autumn | *dispersal* | binary 22/15 23/14 | Trefflich *et al.* 2002 |
| Seed mass | Mean weight of one seed  (in mg) | Mass | germination | numerical | Hintze *et al.* 2013  Otto 2002 |
| Seed number | Seed number per plant: <1.000;  >1.000 (1.000-10.000; >10.000) | seed_nr_01 seed_nr_02 | germination | binary 11/26 26/11 | Kleyer *et al.* 2008  Poschlod *et al.* 2003 |
| Germination season | Season in which plant species germs:  spring;  summer;  autumn | germ_spr germ_su germ_aut | germination | binary 33/4 33/4 27/10 | Fitter & Peat 1994  Hölzel & Otte 2004 |
| Dormancy | Seeds sustain extended periods of unfavourable conditions | dormancy | germination | binary 15/22 | Dr. Monika Partzsch |
| SLA | Specific leaf area: leaf area per dry mass (in mm^2^ mg^-1^) | SLA | competitive ability | numerical | Poschlod *et al.* 2003  Kleyer *et al.* 2008  Engst *et al.* 2016 |
| LDMC | Leaf dry matter content: leaf dry mass per leaf fresh mass  (in mg g^-1^) | LDMC | competitive ability | numerical | Kleyer *et al.* 2008  Breitschwerdt, Jandt & Bruelheide 2015  Engst *et al.* 2016 |
| Plant height | Mean individual height  (in m) | Height | competitive ability | numerical | Kleyer *et al.* 2008  Breitschwerdt, Jandt & Bruelheide 2015  Jäger 2011 |
| Lifeform | Classes of life form:  hemicryptophyte;  geophyte;  therophyte | LF_hemi LF_geo LF_thero | persistence | binary 3/34 31/6 35/2 | Krumbiegel 2002 |
| Age of first flowering | Age (in years) in which species first flowers:  <1;  >1 (1-5, >5) | FF_<1 FF_>1 | persistence | binary 21/16 7/30 | Kleyer *et al.* 2008  Fitter & Peat 1994 |
| Flower duration | Number of months in which the plant flowers | FD | persistence | numerical | Trefflich *et al.* 2002 |
| Clonal growth organ  (CGO) | Classes of CGO’s: epigeal (epigeogenous stem; aboveground runner); hypogeal (hypogeogenous stem; roots with adventitious buds); no | cgo_epi cgo_hyp  cgo_no | persistence | binary 24/13 26/11  25/15 | Klimešová & De Bello 2009 |
| Ecological strategy | Strategy types following Grime (1979):  C;  CSR;  CS & R | ST_C ST_CSR ST_CS_R | persistence | binary 23/14 23/14 28/9 | Klotz & Kühn 2002 |
| Rosette type | Classes of rosette type:  rosette (full rosette; half-rosette);  no rosette | RT_ros RT_no | persistence | binary 13/24 23/14 | Krumbiegel 2002 |

* Further sources for single species were:
Seed number: Marshall and Wilkins 2003, commitment of Prof. Dr. E. Jäger; Germination season: commitment of Prof. Dr. E. Jäger, commitment of M. Stolle, own commitment; Seed shedding season: own commitment; Age of first flowering: commitment of Prof. Dr. E. Jäger, own commitment

References

Breitschwerdt, E., Jandt, U. & Bruelheide, H. (2015). Do newcomers stick to the rules of the residents? Designing trait-based community assembly tests. *Journal of Vegetation Science*, *26*, 219–232.

Engst, K., Baasch, A., Erfmeier, A., Jandt, U., May, K., Schmiede, R. & Bruelheide, H. (2016). Functional community ecology meets restoration ecology: Assessing the restoration success of alluvial floodplain meadows with functional traits. *Journal of Applied Ecology*, *53*, 751–764.

Fitter, A.H. & Peat, H.J. (1994). The Ecological Flora Database. *Journal of Ecology*, *82*, 415–425.

Hintze, C., Heydel, F., Hoppe, C., Cunze, S., König, A. & Tackenberg, O. (2013). D³: The Dispersal and Diaspore Database - Baseline data and statistics on seed dispersal. *Perspectives in Plant Ecology, Evolution and Systematics*, *15*, 180–192.

Hölzel, N. & Otte, A. (2004). Ecological significance of seed germination characteristics in flood-meadow species. *Flora*, *199*, 12–24.

Jäger, E.J. (ed.) (2011). Rothmaler - Exkursionsflora von Deutschland Gefäßpflanzen: Grundband. Spektrum Akademischer Verlag, Heidelberg.

Kleyer, M., Bekker, R.M., Knevel, I.C., Bakker, J.P., Thompson, K., Sonnenschein, M. … Peco, B. (2008). The LEDA Traitbase: a database of life-history traits of the Northwest European flora. *Journal of Ecology*, *96*, 1266–1274.

Klimešová, J. & De Bello, F. (2009). The database of clonal and bud bank traits of Central European flora. *Journal of Vegetation Science*, *20*, 511–516.

Klotz, S. & Kühn, I. (2002). Ökologische Strategietypen. *BIOLFLOR – Eine Datenbank zu biologisch–ökologischen Merkmalen der Gefäßpflanzen in Deutschland* (eds Klotz, S., Kühn, I. & Durka, W.), pp. 197-201. Bundesamt für Naturschutz, Bonn.

Krumbiegel, A. (2002). Morphologie der vegetativen Organe (außer Blätter). *BIOLFLOR – Eine Datenbank zu biologisch–ökologischen Merkmalen der Gefäßpflanzen in Deutschland* (eds Klotz, S., Kühn, I. & Durka, W.), pp. 93–118. Bundesamt für Naturschutz, Bonn.

Marshall, A.H. & Wilkins, P.W. (2003). Improved seed yield in perennial ryegrass (Lolium perenne L.) from two generations of phenotypic selection. *Euphytica*, *133*, 23–241.

Otto, B. (2002). Merkmale von Samen, Früchten, generativen Germinulen und generativen Diasporen. *BIOLFLOR – Eine Datenbank zu biologisch–ökologischen Merkmalen der Gefäßpflanzen in Deutschland* (eds Klotz, S., Kühn, I. & Durka, W.), pp. 177–196. Bundesamt für Naturschutz, Bonn.

Poschlod, P., Kleyer, M., Jackel, A.K., Dannemann, A. & Tackenberg, O. (2003). BIOPOP – A Database of Plant Traits and Internet Application for Nature Conservation. *Folia Geobotanica*, *38*, 263–271.

Trefflich, A., Klotz, S. & Kühn, I. (2002). Blühphänologie. *BIOLFLOR – Eine Datenbank zu biologisch–ökologischen Merkmalen der Gefäßpflanzen in Deutschland* (eds Klotz, S., Kühn, I. & Durka, W.), pp. 127–131. Bundesamt für Naturschutz, Bonn.

**Table S3.** Species × trait matrix for species used in the analyses

**Table S4.** Frequencies of analysed species across both methods (hay, threshing) and both study sites (Küchenholzgraben, Untere Schwarze Elster). In the calculation, we took into account, if a species had the possibility of occurrence in both sites

**Table S5.** ANOVA results of linear mixed effect models for the response of successful establishment of species depending on one predictor variable, method, year and their interactions. The table shows the 24 models that were worse than the no-trait model. For the best 24 models see Table 1. The models are ranked by AIC. Significant effects are indicated as * *P* < 0.05, ** *P* < 0.01, *** P < 0.001, . P < 0.08 and shown in bold fonts. Clonal growth organ: bulb and Flower season: summer are not presented
